# The Status of and Factors Associated With Quiet Quitting Among Clinical Nurses: An Evaluation and Latent Profile Analysis

**DOI:** 10.1155/jonm/2963982

**Published:** 2026-10-21

**Authors:** Haiping Jin, Xiaoling Yang, Jiya Chen, Linghong Mao, Rongrong Wang, Tao-Hsin Tung

**Affiliations:** ^1^ Orthopaedics Taizhou Hospital of Zhejiang Province Affiliated to Wenzhou Medical University Taizhou Zhejiang China wmu.edu.cn; ^2^ Faculty of Nursing Wenzhou Medical University Wenzhou Zhejiang China wmu.edu.cn; ^3^ Hospital Administration Taizhou Hospital of Zhejiang Province Affiliated to Wenzhou Medical University Taizhou Zhejiang China wmu.edu.cn; ^4^ Nursing Department Taizhou Hospital of Zhejiang Province Affiliated to Wenzhou Medical University Taizhou Zhejiang China wmu.edu.cn; ^5^ Evidence-Based Medicine Center Taizhou Hospital of Zhejiang Province Linhai Zhejiang China zjtzyy.h.51daifu.com; ^6^ School of Public Health Hangzhou Medical College Hangzhou Zhejiang China hznu.edu.cn

**Keywords:** associated factors, clinical nurses, latent profile analysis, quiet quitting

## Abstract

**Aims:**

This study aimed to examine the prevalence and latent profiles of quiet quitting among clinical nurses and identify factors associated with this condition across different nurse subgroups.

**Background:**

Quiet quitting among clinical nurses poses a hidden threat to nursing workforce stability and quality of care. Burnout, work–family conflict, and professional identity are closely associated with quiet quitting in nurses. However, academic studies examined quiet quitting as a single global construct, without identifying the potentially heterogeneous profiles within the nursing workforce.

**Methods:**

Convenience sampling was used to recruit clinical nurses employed at a Grade A tertiary hospital in Zhejiang Province between September 4 and 30, 2025. Data were collected using five questionnaires. The components used for the analysis included a general information questionnaire, the Quiet Quitting Scale, Maslach Burnout Inventory, Work–Family Conflict Scale, and Professional Identity Scale. Latent profile analysis (LPA) was used to identify characteristic profiles of quiet quitting among nurses. Multinomial logistic regression analysis was performed to identify the factors associated with the different latent profile categories.

**Results:**

In total, 307 nurses participated in this study. The prevalence of quiet quitting was 76.2%, with an average score of 2.41 ± 0.56 points per item on the quiet‐quitting scale. The LPA identified three distinct quiet‐quitting profiles of nurses: “Conscientious Type” (21.82%), the “Observant Type” (65.15%), and the “Disengaged Type” (13.03%). Multinomial logistic regression analysis showed that burnout, professional identity, and professional title were factors associated with “Observant Type” nurses. In contrast, burnout, work–family conflict, and professional title were identified as factors associated with “Disengaged Type” nurses.

**Conclusions:**

Burnout, work–family conflict, professional identity, and professional title were identified as factors associated with quiet quitting among clinical nurses. Nursing administrators should develop targeted intervention strategies based on the characteristics of each identified nursing profile.

**Implications for Nursing Management:**

Nursing administrators should implement profile‐stratified interventions (psychological support, workload optimization, equitable recognition, and structured professional development) alongside structural empowerment and transparent reward systems to strengthen the professional identity and workforce stability of nursing staff.

## 1. Introduction

Nurses routinely face a wide range of occupational stressors and negative situations, including adverse work environments, work‐related physical and verbal violence, disproportionate workload relative to compensation, high prevalence of burnout, and work–life imbalance. This reality has fueled the growing prevalence of “quiet quitting” (QQ) in the nursing workforce [1, 2]. QQ is defined as a proactive psychological and emotional disengagement from work by employees, characterized by fulfilling only the minimum required duties of their positions and consciously avoiding additional tasks beyond their stipulated responsibilities [3, 4]. This phenomenon not only undermines organizational cohesion and workplace culture but also directly impacts the quality of nursing care, thereby affecting patient safety and reducing satisfaction with healthcare services [5].

Nurses perceive a mismatch between their investments, such as emotional labor, professional effort, time, and organizational returns, including fair compensation. With respect to professional aspects, organizational support, and career development opportunities, the psychological contract between them and the organization is breached and imbalanced [4, 6]. Faced with the perceived high costs of actual turnover and limited alternative employment options, nurses refrain from voluntarily resigning. Instead, they resort to QQ behavior, reducing their work investment as a coping mechanism, so as to restore psychological balance, by maintaining only the minimal level of work engagement necessary to avoid termination [7].

QQ is prevalent among clinical nurses. Multiple studies have reported that the incidence of QQ among nurses ranges from 46.06% to 77.30% [8–10], which is significantly higher than that among other healthcare professionals [11]. Several factors have been identified that contribute to QQ among nurses, including insufficient organizational support [12, 13], excessive workload [14], diminished quality of work life [15], and a lack of moral resilience [8]. However, current research presents some limitations. Most studies have treated the nursing population as a homogeneous group and have employed variable‐centered analytical approaches to investigate the determinants of QQ. This approach has failed to adequately account for the impact of individual differences on the heterogeneity of QQ behaviors. Furthermore, analyses of associated factors often concentrate on single dimensions, such as burnout [12], work engagement [17], or organizational justice and recognition [18], although the behavioral subtypes of QQ arising from the interactive effects of multiple factors have not been identified.

To address the critical influence of population heterogeneity among nurses, this study draws on the Job Demands–Resources (JD‐R) model as a theoretical framework. According to this model, job demands (e.g., work–family conflict) and personal resources (e.g., professional identity) jointly influence burnout levels and behavioral responses of nurses [19]. Furthermore, the model posits that individual differences exist in the combinations of job demands and resources among nurses, which may result in distinct patterns of work–family conflict (job demand), professional identity (personal resources), burnout (health impairment outcome), and QQ (behavioral outcome). To identify these potentially heterogeneous patterns, in this study, we employed a latent profile analysis (LPA), a person‐centered analytical approach. We sought to identify the latent categories underlying QQ behaviors of nurses and to analyze the differences in their demographic and work characteristics across distinct nurse categories [20]. Thus, utilizing the LPA method, this study was designed to examine the potential latent categories of QQ among clinical nurses in a Grade A tertiary hospital and to analyze the population characteristics and factors associated with each category.

## 2. Methods

### 2.1. Study Design

This study employed a cross‐sectional design. Using the convenience sampling method, clinical nurses working at a Grade A tertiary hospital in Zhejiang Province were recruited between September 4 and 30, 2025. A total of 312 questionnaires were collected, of which 307 were considered valid responses (effective response rate: 98.4%). The inclusion criteria required the participants to possess a valid nurse practicing certificate registered with the hospital. The exclusion criteria were as follows: (1) nurses assigned to nonclinical departments (e.g., outpatient clinics and medical technology departments) and (2) those with cumulative leave (due to sickness, maternity, or personal reasons) exceeding 3 months within the preceding 3‐month period. The study included 21 independent variables. The sample size was calculated based on Kendall’s principle, which recommends a minimum of 10 participants per independent variable [21]. Considering a 20% ineffective response rate, the estimated sample size was 263 participants. For LPA, current recommendations based on Monte Carlo simulations suggested that a total sample of 300–500 participants were generally sufficient for stable model convergence and accurated class assignment when the smallest class proportion was above 10% [22]. Accordingly, we included 307 participants, which met the recommended threshold for LPA.

The study was approved by the Ethics Committee of Taizhou Hospital in Zhejiang Province (Approval No. K20250905 [EZ]) and strictly adhered to the principles of the Declaration of Helsinki. All the participants provided informed consent and participated voluntarily.

### 2.2. Measures

#### 2.2.1. General Information Questionnaire

The general information questionnaire was designed by the research team and was used to collect data on the following variables: sex, age, years of work experience, educational background, professional title, employment type, monthly income level, marital status, parental status, shift‐work status, and number of weekly night shifts.

#### 2.2.2. Quiet Quitting Scale (QQS)

This study utilized the Chinese version of the QQS. The original scale was developed by Galanis et al. in a Greek employee sample [23]. The scale was subsequently translated into Chinese, and its psychometric properties were validated by domestic researchers [24]. It consists of three dimensions: emotional detachment, lack of initiative, and lack of motivation. A cutoff value of 2.06 was adopted to identify quiet quitters [8]. Cronbach’s *α* for the scale was 0.856. In the present study, Cronbach’s *α* was 0.774.

#### 2.2.3. Maslach Burnout Inventory (MBI)

Burnout was assessed using the MBI originally developed by Maslach and Jackson in 1981 [26]. The Chinese version, translated and adapted by Li and Liu, consists of 22 items measuring three dimensions: emotional exhaustion, depersonalization, and reduced personal accomplishment [27]. Higher MBI scores indicate more severe burnout. The reported Cronbach’s *α* for the Chinese version was 0.93 [27]. In this study, Cronbach’s *α* was 0.938.

#### 2.2.4. Work–Family Conflict Scale (WFCS)

The WFCS, originally developed by Netemeyer et al. [28], was used to measure work–family conflict. The Chinese version was translated and validated by domestic researchers [29] and consists of two dimensions: work‐to‐family and family to‐work conflicts. Higher scores indicate a greater degree of conflict. The scale demonstrated Cronbach’s *α* coefficients ranging from 0.90 to 0.93 in a previous validation study [29]. In this study, Cronbach’s *α* was 0.890.

#### 2.2.5. Professional Identity Scale (PIS) for Nurses

This study used the PIS for nurses developed by Liu et al. [30]. This scale comprises 30 items and encompasses 5 dimensions: professional cognition, professional social support, professional social skills, coping with occupational setbacks, and professional self‐reflection. A higher total score indicated a stronger level of professional identity among the nurses. The scale demonstrated a Cronbach’s *α* coefficient of 0.938 in the original study [30]. In the present study, Cronbach’s *α* was 0.972.

### 2.3. Data Collection and Quality Control

Data were collected using a star questionnaire platform. Approval from the hospital’s nursing department and head nurses of each clinic department was obtained, and the questionnaire was distributed to clinical nurses by the head nurses. Nurses who met the inclusion criteria were informed of the study’s purpose and invited to participate voluntarily. The platform was set to allow only one response per device and one IP address, and all items had to be completed in advance prior to submission. The first page of the questionnaire featured a standardized instruction section that clearly outlined the purpose of the study, confidentiality guarantees, and completion guidelines. It also included a consent item for participation in the survey; selecting “Agree” granted access to the questionnaire, whereas selecting “Disagree” resulted in immediate exit from the survey. Submitted responses were directly stored in the back end of the platform for storage and analysis. After data export, a two‐person team independently verified and entered data to ensure accuracy and completeness.

### 2.4. Common Method Bias Test

Because all the data were collected from the same source and context, common method bias could be a potential concern [31]. To control for this bias, procedural remedies were implemented a priori, including ensuring respondents’ anonymity, incorporating reverse‐coded items, and emphasizing that there were no correct or incorrect answers. Subsequently, a post hoc Harman’s single‐factor test was conducted. An unrotated exploratory factor analysis extracted 12 factors with eigenvalues > 1. The first factor accounted for 38.06% of the total variance, which is below the recommended threshold of 40%. Combined with a priori procedural controls, these results suggest that the common method bias is unlikely to be a serious concern.

### 2.5. Statistical Analyses

This study employed SPSS 29.0 and Mplus 6.12 for statistical analyses. Categorical variables were described as frequencies and percentages and continuous variables as means ± standard deviations. Between‐profile differences were tested using one‐way ANOVA, chi‐square tests, or Kruskal–Wallis H tests depending on variable type and distribution. Variables with *p* < 0.05 in univariate analyses were entered into multinomial logistic regression, with the Conscientious Type as the reference category. The assumptions of multinomial logistic regression were checked, including mutual exclusivity and exhaustiveness of outcome categories and independence of observations. Multicollinearity was assessed using variance inflation factors (VIF > 10 indicating collinearity) [32].

LPA was conducted using the nine items of the QQS as continuous indicators, with models containing an increasing number of profiles estimated sequentially. Model fit was evaluated using the Akaike information criterion (AIC), Bayesian information criterion (BIC), and sample‐size‐adjusted Bayesian information criterion (aBIC), entropy, the Lo–Mendell–Rubin adjusted likelihood ratio test (LMRT), the bootstrapped likelihood ratio test (BLRT), and profile size. Lower values of AIC, BIC, and aBIC indicated a better model fit. The entropy value ranges from 0 to 1, with a value ≥ 0.80 indicating acceptable classification accuracy. The LMRT and BLRT were used to compare the fit between the *k*‐class and (*k*−1)‐class models. A *p*‐value < 0.05 for these tests indicated that the *k*‐class model provided a significantly better fit than the (*k*−1)‐class model. The smallest class should typically comprise no less than 5% of the total sample. The optimal model was selected based on statistical fit, classification quality, interpretability, and profile stability.

## 3. Results

### 3.1. Participant Characteristics

As shown in Figure 1, the implementation process identified 234 nurses who met QQ criteria, representing 76.2% of the sample. This classification was based on the proposed cutoff value of 2.06 [8], which has not been validated in Chinese nurse populations. The ages of 307 participating nurses ranged from 20 to 53 years, with a mean of 32.41 ± 6.92 years. The cohort comprised 14 males (4.6%) and 293 females (95.4%). Their work experience ranged from 0 to 33 years, with a mean of 10.65 ± 7.52 years. Approximately 85.3% of the participants had a bachelor’s degree or higher. With respect to professional titles, 56.0% held junior‐level status and 44.0% held intermediate professional title or above. A total of 61.6% were employed as permanent staff. Regarding marital status, 65.8% of participants were married (of whom 58.3% had children) and 34.2% were unmarried. Monthly income was distributed with 14.7% earning below 5000 RMB and 85.3% earning 5000 RMB or above. Shift work was prevalent, involving 89.9% of the cohort, versus 10.1% who did not engage in shift work.

**FIGURE 1:**
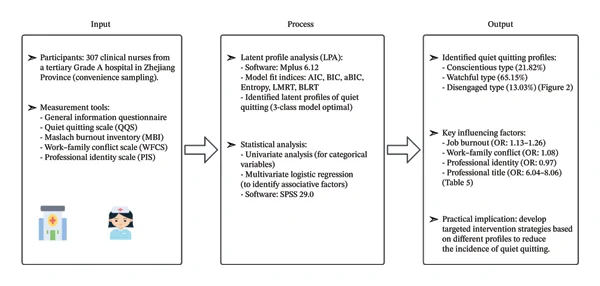
The research design and implementation process.

### 3.2. LPA on Nurse QQ

The average QQS score of the nurses (*n* = 307) was 2.41 ± 0.56. The dimension scores for emotional detachment, lack of initiative, and lack of motivation were 2.53 ± 0.6, 2.24 ± 0.7, and 2.44 ± 0.77, respectively. Using the scores from the nine items in the QQS as manifest variables, latent class models comprising 1–4 profiles were constructed sequentially. The model fit indices are presented in Table 1.

**TABLE 1:** Fit indices of the latent profile model for nurse quiet quitting (*n* = 307).

Model category	AIC	BIC	aBIC	Entropy	*p* (LMRT)	*p* (BLRT)	Category probability
Class 1	7392.136	7459.220	7402.132	1.000	—	—	—
Class 2	6995.940	7100.292	7011.488	0.773	0.001	< 0.001	0.57/0.43
Class 3	6832.448	6974.068	6853.549	0.851	0.019	< 0.001	0.22/0.65/0.13
Class 4	6721.068	6899.957	6747.722	0.882	0.340	< 0.001	0.02/0.14/0.24/0.60

As the number of profiles increased, the AIC, BIC, and aBIC progressively decreased. For the three‐profile solution, the *p*‐value of the LMRT was 0.019, indicating a significantly better model fit compared to the two‐profile solution. However, the four‐profile solution yielded a nonsignificant *p*‐value (*p* = 0.340), suggesting no substantial improvement in model fit. The entropy value for the three‐profile solution was 0.851, indicating high classification accuracy and low classification uncertainty. Therefore, based on these results and comprehensive evaluation of the fit, the three‐profile model was selected as the optimal solution. Each participant was then assigned to the profile with the highest posterior probability (i.e., modal class assignment) for subsequent multinomial logistic regression analyses.

Figure 2 illustrates the score distribution of the three latent profiles across the nine QQS items. Profile C1, characterized by consistently low scores on emotional detachment, lack of initiative, and lack of motivation, was labeled the “Conscientious Type,” accounting for 21.82% of the sample (*n* = 67). Profile C2 exhibited moderate scores across all three dimensions and was therefore designated the “Observant Type,” representing the largest proportion at 65.15% (*n* = 200). Profile C3 showed the highest scores on all three dimensions and was classified as the “Disengaged Type,” comprising 13.03% of the sample (*n* = 40). These three profiles formed a meaningful severity gradient (C1 < C2 < C3), which was further corroborated by significant associations with external validating variables—including burnout, work–family conflict, and professional identity (presented in Sections 3.3 and 3.4)—with C1 demonstrating the most favorable outcomes and C3 the least favorable.

**FIGURE 2:**
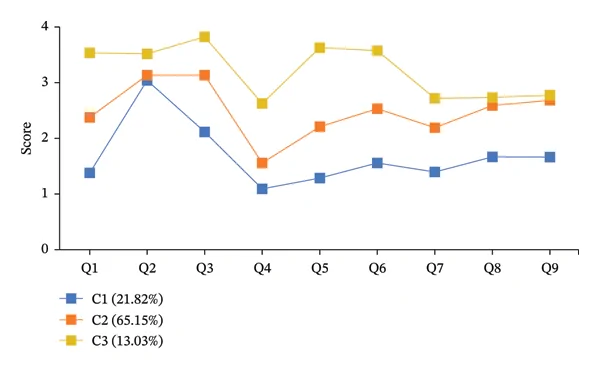
Score characteristics of different potential profile classifications for each entry.

### 3.3. Univariate Analysis of Predictors Across QQ Profiles

The results of the univariate analysis across the three latent profiles revealed significant differences among the profile groups in terms of age (*F*_(2, 304)_ = 26.009, *p* < 0.001), years of work experience (*H*_(4)_, *p* < 0.001), professional title ($\chi_{\left(2\right)}^{2}$ = 41.151, *p* < 0.001), employment type ($\chi_{\left(2\right)}^{2}$ = 17.563, *p* < 0.001), monthly income ($\chi_{\left(2\right)}^{2}$ =  8.060, *p* = 0.018), marital status ($\chi_{\left(2\right)}^{2}$ = 12.282, *p* = 0.002), parental status ($\chi_{\left(2\right)}^{2}$ =  2.629, *p* < 0.001), MBI score (*F*_(2, 304)_ = 105.20, *p* < 0.001), WFCS score (*F*_(2, 304)_ = 30.33, *p* < 0.001), and PIS score (*F*_(2, 304)_ = 59.35, *p* < 0.001). In contrast, no significant differences were observed in terms of sex ($\chi_{\left(2\right)}^{2}$ = 1.510, *p* = 0.470), education ($\chi_{\left(2\right)}^{2}$ = 3.973, *p* = 0.137), shift work system ($\chi_{\left(2\right)}^{2}$ = 5.725, *p* = 0.057), or number of night week shifts ($\chi_{\left(2\right)}^{2}$ = 1.042, *p* = 0.594). For the detailed results, please refer to Tables 2 and 3.

**TABLE 2:** Univariate analysis of nurse quiet quitting potential profiles and general characteristics of survey participants (*n* = 307).

Item	Classification	Total	Conscientious type (*n* = 67)	Observant type (*n* = 200)	Disengaged type (*n* = 40)	Test statistics	*p*
Age (years)		307	37.28 ± 6.78	31.35 ± 6.41	29.55 ± 5.79	*F = *26.009^a^	< 0.001

Sex	Male	14	4	7	3	*χ* ^2^ * = *1.510^b^	0.470
	Female	293	63	193	37		

Years of work experience (years)	≤ 5	95	6	69	20	*H* = 42.203^c^	< 0.001
	6–10	93	13	67	13		
	> 10	119	48	64	7		

Educational background	Specialty and below	45	5	32	8	*χ* ^2^ * = *3.973^b^	0.137
	Bachelor’s degree or higher	262	62	168	32		

Professional title	Junior professional title	172	15	127	30	*χ* ^2^ * = *41.151^b^	< 0.001
	Intermediate professional title or above	135	52	73	10		

Employment type	Contract system	118	11	89	18	*χ* ^2^ * = *17.563^b^	< 0.001
	Official establishment	189	56	111	22		

Monthly income (RMB)	< 5000	45	3	33	9	*χ* ^2^ * = *8.060^b^	0.018
	≥ 5000	262	64	167	31		

Marital status	Unmarried	105	11	77	17	*χ* ^2^ * = *12.282^b^	0.002
	Married	202	56	123	23		

Parental status (person)	No children	128	12	92	24	*χ* ^2^ * = *22.629^b^	< 0.001
	Have children	179	55	108	16		

Shift work status	No	31	12	17	2	*χ* ^2^ * = *5.725^b^	0.057
	Yes	276	55	183	38		

Number of weekly night shifts (times)	≤ 1	206	48	133	25	*χ* ^2^ * = *1.042^b^	0.594
	≥ 2	101	19	67	15		

^a^One‐way ANOVA.

^b^Chi‐square test.

^c^Kruskal–Wallis test.

**TABLE 3:** Associations between different latent profiles and the burnout, work–family conflict, and professional identity scales (*n* = 307).

Scale scores	Mean ± SD	Conscientious type (*n* = 67)	Observant type (*n* = 200)	Disengaged type (*n* = 40)	*F*	*p*
MBI score	53.04 ± 12.69	39.01 ± 10.15	55.29 ± 10.16	65.25 ± 6.76	105.20	< 0.001
WFCS score	33.34 ± 10.42	26.96 ± 10.90	33.82 ± 9.36	41.63 ± 7.83	30.33	< 0.001
PIS score	103.27 ± 19.61	122.52 ± 17.2	98.93 ± 17.01	92.75 ± 13.9	59.35	< 0.001

### 3.4. Multivariate Logistic Regression Analysis of QQ Profiles

Before conducting the multinomial logistic regression, multicollinearity diagnostics were performed. All VIF values were below 10 (range: 1.173–5.689), and all tolerance values were above 0.10 (range: 0.176–0.853), indicating the low multicollinearity among the independent variables. Profile membership was incorporated using modal class assignment. Classification uncertainty was not directly modeled in the regression; however, the entropy value for the three‐profile solution was 0.851, indicating high classification accuracy and low classification uncertainty. Variables that showed significant differences in the univariate analysis were included as independent variables in the multinomial logistic regression model. The three latent profiles served as the dependent variable, and the “Conscientious Type” served as the reference category. Table 4 presents the assignment of the variables. The scores on the MBI (odds ratio [OR], 1.13–1.26; 95% confidence interval [CI], 1.06–1.37; *p* < 0.001), WFCS (OR, 1.08; 95% CI, 1.01–1.15; *p* = 0.037), PIS (OR, 0.97; 95% CI, 0.94–0.99; *p* = 0.017), and professional title (OR, 6.04–8.06; 95% CI, 1.76–46.59; *p* = 0.004, 0.020) significantly influenced latent profiles (Table 5).

**TABLE 4:** Variable assignment.

Characteristic variable	Characteristic variable assignment
Professional title	Junior professional title = 0, Intermediate professional title or above = 1
Employment type	Contract system = 0, Official establishment = 1
Marital status	Unmarried = 0, married = 1
Parental status	No children = 0, have children = 1
Age (in years)	Substitute the original value in.
Working life	Substitute the original value in.
Monthly income level	Substitute the original value in.
MBI scale	Substitute the original value in.
WFCS scale	Substitute the original value in.
PIS scale	Substitute the original value in.

**TABLE 5:** Multiple logistic regression analysis of different potential profiles.

Variables	Observant type vs. conscientious type			Disengaged type vs. conscientious type		
	*β*	*p*	OR (95% CI)	*β*	*p*	OR (95% CI)
MBI score	0.118	< 0.001	1.13 (1.06, 1.19)	0.230	< 0.001	1.26 (1.16, 1.37)
WFCS score	−0.003	0.911	1.00 (0.95, 1.05)	0.073	0.037	1.08 (1.01, 1.15)
PIS score	−0.035	0.017	0.97 (0.94, 0.99)	−0.020	0.376	0.98 (0.94, 1.03)
Age (years)	0.046	0.412	1.05 (0.94, 1.17)	0.096	0.306	1.10 (0.92, 1.32)
Years of work experience (years)	−0.227	0.707	0.80 (0.24, 2.61)	−1.011	0.215	0.36 (0.07, 1.80)
Professional title (junior vs. intermediate or above)	1.799	0.004	6.04 (1.76, 20.74)	2.087	0.020	8.06 (1.40, 46.59)
Nature of appointment (contract vs. permanent)	0.197	0.749	1.22 (0.36, 4.08)	−0.168	0.825	0.85 (0.19, 3.77)
Monthly income (RMB)	0.906	0.244	2.48 (0.54, 11.38)	1.360	0.143	3.90 (0.63, 24.00)
Marital status (unmarried vs. married)	−0.395	0.681	0.67 (0.10, 4.42)	−1.143	0.308	0.32 (0.04, 2.87)
Parental status (person) (no kids vs. have kids)	0.510	0.589	1.67 (0.26, 10.56)	1.218	0.283	3.38 (0.37, 31.21)

## 4. Discussion

Using LPA, this study identified three distinct types of QQ among clinical nurses: conscientious, observant, and disengaged. These profiles highlight the significant heterogeneity of QQ manifestations in this population. The main factors associated with QQ profiles included burnout, work–family conflicts, professional identity, and professional title.

LPA has been employed to identify a “quiet quitter” profile (23.1%) among healthcare workers, suggesting that this profile could be interpreted as either commitment erosion or adaptive boundary‐setting [33]. However, their profile indicators were teamwork climate, job satisfaction, and emotional exhaustion. QQ was inferred rather than directly measured, and thus, the two interpretations could not be distinguished [34]. This study directly incorporated the QQ construct into LPA, identifying three profiles—conscientious, observant, and disengaged—thereby enabling the differentiation of these two interpretations at the QQ construct level.

This study revealed that 76.2% of the nurses were categorized as experiencing QQ, a finding consistent with previous studies [8, 35]. This high proportion suggests that QQ is a widespread phenomenon among nurses rather than a context‐specific issue. Notably, the “Emotional Detachment” subscale recorded the highest average score among the dimensions. This may be associated with personal values, as the nursing population often places great importance on self‐actualization, personal growth, fairness, compensation, and a positive work environment [36]. This finding diverges from that of previous studies where the “Lack of Motivation” subscale typically scored the highest. The observed discrepancy may indicate a declining trend in intrinsic work motivation among nurses, potentially attributable to perceptions of unfair reward distribution, which reduces their drive to engage in nursing work [36]. From the perspective of the JD‐R model [37], insufficient organizational support and limited professional development opportunities are consistent with the characteristics of the health impairment pathway, manifesting as higher levels of burnout and emotional detachment, accompanied by QQ. Work–family conflict is associated with higher burnout, whereas professional identity is associated with lower burnout. The high scores on the emotional detachment scale may reflect the tendency of nurses to resort to psychological withdrawal as a coping response when their expectations (e.g., self‐actualization, fair compensation, and a positive work environment) are not met [37].

The three distinct types of clinical nurses identified in this study exhibited significant differences in their core influencing factors. Nurses categorized as the “Observant Type” demonstrated low levels of work–family conflict and relatively high professional identity, yet they reported elevated levels of burnout [38]. This can be attributed to several reasons. Although a strong professional identity typically enhances intrinsic work motivation and psychological resources, thereby fostering greater work engagement [39], this protective effect may be undermined by specific contextual factors. One potential explanation is that the “Observant Type” nurses often hold junior‐level professional titles and face limited career development opportunities, which have been associated with increased burnout [40]. Furthermore, systemic stressors, such as unfavorable working conditions, excessive workload, and low wages, have been identified as contributors to emotional exhaustion among healthcare professionals [41]. The combined factors and limited career progression coupled with high workload and low compensation may create an effort–reward imbalance for “Observant Type” nurses and explain their elevated burnout despite strong professional identity.

Nurses classified as the “Disengaged Type” exhibited higher levels of burnout, more severe work–family conflict, and lower professional identity compared with the “Observant Type.” Accumulation of discontent among nurses exacerbates burnout, leading to a progressive buildup of emotional detachment and apathy [39]. Concurrently, work–family conflict directly threatened the physical and mental health status of nurses, which in turn leads to diminished work motivation, decreased performance, reduced job satisfaction, and increased turnover intention, and it may even adversely affect their self‐perceived quality of nursing care [42]. It is noteworthy that these negative effects are more pronounced among “Disengaged Type” nurses. In contrast, “Observant Type” nurses experiencing moderate levels of conflict and burnout demonstrated relatively milder negative manifestations.

### 4.1. Implications for Nursing Management

To ensure the stability of the nursing workforce and improve work conditions, nursing administrators should establish multidimensional and targeted interventions and support mechanisms based on a stratified approach tailored to different QQ profiles. Nurses identified as the “Disengaged Type,” who exhibit severe emotional exhaustion and detachment from work, present critical and complex management challenges. Thus, proactive identification of these nurses is essential. These nurses should be prioritized toward one‐on‐one psychological counseling and emotional support services, such as the introduction of an employee assistance program by employers. This would reduce their levels of emotional exhaustion and depersonalization while enhancing psychological resilience and work engagement [43]. Workload reassessments were conducted concurrently for this group. This may involve appropriate reduction in duties or temporary transfers to less‐stressful work positions, which may help lower the risk of nurses experiencing complete burnout or quitting their jobs.

For the “Observant Type,” management efforts to rebuild a sense of fairness and hope may include (a) publishing clear criteria for performance evaluations and promotions, (b) applying consistent rules for shift assignments and overtime distribution, and (c) formally recognizing extra‐role contributions (e.g., volunteering for difficult shifts or mentoring new staff). These steps could help them feel more tangibly rewarded. For the “Conscientious Type,” management efforts focused on protection and motivation may include (a) offering public recognition of their reliability, (b) providing access to training and professional development programs, and (c) monitoring workload levels through regular check‐ins to prevent silent overcommitment. These strategies might reinforce their professional identity while reducing burnout risk [44]. Finally, nursing administrators could implement broader organizational strategies, such as structural empowerment and enhanced participation in decision‐making processes. These measures may help improve nurses’ sense of control over their work and perceived value. When complemented by a well‐designed reward system (e.g., a transparent, points‐based recognition system), such approaches could strengthen nurses’ sense of professional honor and elevate their overall professional identity.

### 4.2. Study Limitations

This study has several limitations that should be acknowledged. First, the cross‐sectional study design based on data collected at a single time point precluded the ability to infer causal relationships. Although statistical analyses identified significant associations between factors such as occupational burnout, work–family conflict, and QQ profiles, the temporal sequence and direction of these relationships could not be determined. Reverse causality may also exist (e.g., QQ may increase work–family conflict or reduce professional identity). Second, the use of convenience sampling from a single tertiary hospital in Zhejiang Province may have introduced selection bias, which limits the generalizability of the findings. Therefore, these results may not be fully applicable to nurses working in other regions, in different hospital levels, or within distinct cultural and healthcare contexts. Future studies should employ multicenter, randomized sampling strategies to enhance the representativeness and external validity of the results. Third, all data were obtained through nurse self‐report questionnaires, which is susceptible to reporting biases, such as social desirability or recall bias. Despite assurances of anonymity and confidentiality, the effect of these potential biases cannot be ruled out. Future research would benefit from incorporating objective measures or mixed‐method approaches to triangulate and validate the findings. Fourth, the categorization of QQ relied on a proposed cutoff value of 2.06. Considering that this threshold has not been widely applied in the context of Chinese nursing, further research is required to explore its relevance and applicability in this setting. Fifth, although Harman’s single‐factor test indicated that common method bias was unlikely to be a serious concern, the use of a single time point and self‐reported data may introduce potential bias. Sixth, although our sample size (*n* = 307) met the minimum recommendation of 300 for LPA according to Kendall’s principle, the methodological guidelines indicated that a sample size > 500 participants is required to obtain reliable classification and parameter estimates. Future studies with larger sample sizes are required to validate our findings. Seventh, because the Disengaged group was relatively small (*n* = 40), some estimates had wider CIs; findings involving this group should therefore be interpreted as exploratory and require replication in larger samples. Finally, the use of modal class assignment may introduce classification uncertainty, as it treats profile membership as perfectly observed and does not account for posterior probabilities. However, the entropy value for the three‐profile solution was 0.851, indicating high classification accuracy and low classification uncertainty.

## 5. Conclusions

This study identified three distinct QQ subtypes among clinical nurses: conscientious, observant, and disengaged. Burnout, work–family conflict, professional identity, and professional title were significantly associated with distinguishing these latent QQ profiles. Therefore, it is imperative to develop nursing management featuring multidimensional and targeted interventions and support systems. These strategies should adopt a stratified approach to address the specific characteristics of each unique QQ profile among nursing staff.

## Author Contributions

Haiping Jin: conceptualization, formal analysis, data curation, and writing–original draft. Xiaoling Yang: methodology, investigation, and data curation. Jiya Chen: validation and writing–review and editing. Linghong Mao: investigation and writing–review and editing. Rongrong Wang: supervision, funding acquisition, and writing–review and editing. Tao‐Hsin Tung: conceptualization, resources, methodology, and writing–review and editing.

## Funding

This work was supported by the National Natural Science Foundation of China (funding ID 72374157).

## Ethics Statement

The study was approved by the Ethics Committee of Taizhou Hospital in Zhejiang province (Approval No: K20250905 [EZ]) and strictly adhered to the principles of the Declaration of Helsinki.

## Conflicts of Interest

The authors declare no conflicts of interest.

## Data Availability

The anonymized dataset used and/or analyzed during the current study is available from the corresponding author upon reasonable request, subject to ethical and institutional requirements.

## References

[1] Boy Y. and Sürmeli M. , Quiet Quitting: A Significant Risk for Global Healthcare, Journal of Global Health. (2023) 13, 10.7189/jogh.13.03014. PMC10062397 36995298

[2] Ng I. K. , Goh W. G. , Thong C. , and Teo K. S. , Quiet Quitting Among Medical Practitioners: A Hallmark of Burnout, Disillusionment and Cynicism, Journal of the Royal Society of Medicine. (2025) 118, no. 3, 73–77, 10.1177/01410768241311059. PMC11826815 39946171

[3] Arneja J. S. and Gilardino M. S. , Quiet Quitting: Why is It Worse in Healthcare and What do We do About It?, Plastic Surgery. (2023) 31, no. 4, 319–320, 10.1177/22925503231208495. PMC10617448 37915354

[4] Patel P. C. , Guedes M. J. , Bachrach D. G. , and Cho Y. , A Multidimensional Quiet Quitting Scale: Development and Test of a Measure of Quiet Quitting, PLoS One. (2025) 20, no. 4, 10.1371/journal.pone.0317624. PMC11999150 40233122

[5] Kang J. , Kim H. , and Cho O.-H. , Quiet Quitting Among Healthcare Professionals in Hospital Environments: A Concept Analysis and Scoping Review Protocol, BMJ Open. (2023) 13, no. 11, 10.1136/bmjopen-2023-077811. PMC10660974 37984954

[6] Shah M. K. , Gandrakota N. , Cimiotti J. P. , Ghose N. , Moore M. , and Ali M. K. , Prevalence of and Factors Associated With Nurse Burnout in the US, JAMA Network Open. (2021) 4, no. 2, 10.1001/jamanetworkopen.2020.36469. PMC7862989 33538823

[7] Kang J. , Jeong W. , and Kim S. , The Mediating Effects of COVID-19 Infection Control Fatigue on Quiet Quitting: Focusing on Organisational Justice, Role Ambiguity and Job Satisfaction, Journal of Advanced Nursing. (2025) 81, no. 7, 3952–3961, 10.1111/jan.16865. PMC12159379 40035260

[8] Galanis P. , Katsiroumpa A. , Vraka I. et al., Quiet Quitting Among Employees: A Proposed Cut-Off Score for the Quiet Quitting Scale, Archives of Hellenic Medicine. (2024) 41, no. 3, 381–387, https://www.mednet.gr/archives/2024-3/pdf/381.pdf.

[9] Gün İ. , Kutun F. Ç. , and Söyük S. , Mediating Effect of Turnover Intention on the Relationship Between Job Burnout and Quiet Quitting in Nurses, Journal of Advanced Nursing. (2025) 81, no. 10, 6569–6577, 10.1111/jan.16807. PMC12460954 39930081

[10] Rinaldi S. and Pomarolli E. , Quiet Quitting Among Nurses: A Case Study in a Northern Italian Hospital, Nursing Reports. (2025) 15, no. 7, 10.3390/nursrep15070239. PMC12300128 40710934

[11] Galanis P. , Katsiroumpa A. , Vraka I. et al., Nurses Quietly Quit Their Job More Often Than Other Healthcare Workers: An Alarming Issue for Healthcare Services, International Nursing Review. (2021) 71, no. 4, 850–859, 10.1111/inr.12931. 38193567

[12] Gün İ. , Balsak H. , and Ayhan F. , Mediating Effect of Job Burnout on the Relationship Between Organisational Support and Quiet Quitting in Nurses, Journal of Advanced Nursing. (2024) 81, no. 8, 4644–4652, 10.1111/jan.16599. PMC12271679 39498691

[13] Geng R. , Geng X. , and Geng S. , Identifying Key Antecedents of Quiet Quitting Among Nurses: A Cross-Profession Meta-Analytic Review, Journal of Advanced Nursing. (2025) 82, no. 1, 913–933, 10.1111/jan.16934. 40167291

[14] Galanis P. , Moisoglou I. , Katsiroumpa A. et al., Workload Increases Nurses’ Quiet Quitting, Turnover Intention, and Job Burnout: Evidence From Greece, AIMS Public Health. (2025) 12, no. 1, 44–55, 10.3934/publichealth.2025004. PMC11999808 40248406

[15] Stankovic M. and Slavkovic M. , Quality of Work Life Determinants of Healthcare Professionals’ Quiet Quitting: Towards Individual Difference, Healthcare. (2025) 13, no. 13, 10.3390/healthcare13131547. PMC12248897 40648572

[16] Galanis P. , Moisoglou I. , Katsiroumpa A. et al., Impact of Workplace Bullying on Quiet Quitting in Nurses: The Mediating Effect of Coping Strategies, Healthcare. (2022) 12, no. 7, 10.3390/healthcare12070797. PMC11011316 38610219

[17] Moisoglou I. , Katsiroumpa A. , Papathanasiou I. V. et al., Engaging Leadership Reduces Quiet Quitting and Improves Work Engagement: Evidence From Nurses in Greece, Nursing Reports. (2025) 15, no. 7, 10.3390/nursrep15070247. PMC12298008 40710942

[18] Toska A. , Dimitriadou I. , Togas C. et al., Quiet Quitting in the Hospital Context: Investigating Conflicts, Organizational Support, and Professional Engagement in Greece, Nursing Reports. (2025) 15, no. 2, 10.3390/nursrep15020038. PMC11858196 39997774

[19] Montenegro Méndez S. , Laguía González A. , and Moriano León J. A. , Is Leadership a Resource? A Systematic Review of Its Role in Burnout and Engagement Among Nurses Within the JD-R Model, Journal of Nursing Management. (2025) 2025, no. 1, 10.1155/jonm/8853148. PMC12677997 41355851

[20] Chen Y. , Li Y. , Zhang J. et al., Subgroups of Relational Job Characteristics and Their Differences in Turnover Intention and Subjective Well-Being Among Nurses: A Latent Profile Analysis, Biomedical Chromatography. (2024) . 10.1186/s12912-024-02141-2 PMC11229294 38977977

[21] Jin M. , Qian R. , Wang J. et al., Influencing Factors Associated With Mental Workload Among Nurses: A Latent Profile Analysis, International Journal of Nursing Science. (2024) 11, no. 3, 330–337, 10.1016/j.ijnss.2024.04.002. PMC11329065 39156683

[22] Nylund-Gibson K. and Choi A. Y. , Ten Frequently Asked Questions About Latent Class Analysis, Translational Issues in Psychological Science. (2018) 4, no. 4, 440–461, 10.1037/tps0000176.

[23] Galanis P. , Katsiroumpa A. , Vraka I. et al., The Quiet Quitting Scale: Development and Initial Validation, AIMS Public Health. (2023) 10, no. 4, 828–848, 10.3934/publichealth.2023055. PMC10764970 38187899

[24] Zhang D. , Zheng H. , Zeng Y. et al., Psychometric Properties of the Chinese Version of the Quiet Quitting Scale, BMC Nursing. (2025) 24, no. 1, 10.1186/s12912-025-02921-4. PMC11900426 40069793

[25] Galanis P. , Moisoglou I. , Katsiroumpa A. et al., Moral Resilience Reduces Levels of Quiet Quitting, Job Burnout, and Turnover Intention Among Nurses: Evidence in the Post COVID-19 Era, Nursing Reports. (2020) 14, no. 1, 254–266, 10.3390/nursrep14010020. PMC10885038 38391065

[26] Maslach C. and Jackson S. E. , The Measurement of Experienced Burnout, Journal of Occupational Behaviour. (1981) 2, 99–113, 10.1002/job.4030020203.

[27] Li X. and Liu Y. , Job Stressors and Burnout Among Staff Nurses, Chinese Journal of Nursing. (2000) 35, no. 11, 645–649.

[28] Netemeyer R. G. , Boles J. S. , and McMurrian R. , Development and Validation of Work-Family Conflict and Family-Work Conflict Scales, Journal of Applied Psychology. (1996) 81, no. 4, 400–410, 10.1037/0021-9010.81.4.400.

[29] Ge P. , Yang Y. , Liu S. et al., Validity and Reliability of the Chinese Version of the Work-Family Conflict Scale in Working Parents, Chinese Mental Health Journal. (2023) 37, no. 2, 145–150, 10.3969/j.issn.1000-6729.2023.02.008.

[30] Liu L. , Hao Y. , and Liu X. , Development of Professional Identity Scale for Nurses, Nursing Journal of Chinese People’s Liberation Army. (2011) 28, no. 3, 18–20, 10.3969/j.issn.1008-9993.2011.03.006.

[31] Qin Z. , Zhang H. , Su S. et al., Perceived Professional Preparedness and Identity Among Senior Nursing Students: A Latent Profile Analysis, BMC Nursing. (2024) 23, no. 1, 10.1186/s12912-024-01965-2. PMC11057085 38684995

[32] Li C. , Lu X. , Zhang L. et al., Latent Profile Analysis of Nurses’ Knowledge, Attitudes, and Practices Regarding Pressure Injury Prevention: A Multicenter Large-Sample Study, BMC Nursing. (2025) 24, no. 1, 10.1186/s12912-025-03875-3. PMC12482050 41024115

[33] Chang Y.-C. , Lin G.-M. , Wu C.-H. , and Wang Y.-C. , A 6-Year Latent Profile Analysis of Teamwork Climate, Job Satisfaction, and Emotional Exhaustion Among Healthcare Workers at a Taiwanese Medical Center, Nursing Outlook. (2026) 74, no. 4, 10.1016/j.outlook.2026.102839. 42349367

[34] Cesaro E. and Daicampi C. , Quiet Quitting in the Healthcare Workforce: A Systematic Review of Organisational Drivers and Managerial Implications, Journal of Nursing Management. (2026) 2026, no. 1, Advance online publication, 10.1155/jonm/7319117. PMC13392511 42487465

[35] Othman A. A. , Mahran H. M. , and Ali H. I. , Covering Loyalty Policy in Quiet Firing Workplace: The Association Between Quiet Quitting, Intention to Leave, and Nurses’ Loyalty, BMC Nursing. (2025) 24, no. 1, 10.1186/s12912-025-03301-8. PMC12181833 40542367

[36] Yiu T.-F. , Huang Y.-C. , and Wang K.-Y. , The Quiet Quitting in Nursing Workplaces and Coping Strategies, Journal of Nursing. (2025) 72, no. 3, 95–101, 10.6224/JN.202506_72(3).12. 40419433

[37] Richard E. , Metersky K. , Matthews L. , and MacLean R. , Understanding the Work Life of Clinical Nurse Educators in Canadian Hospitals Through the Job Demands-Resources Model: A Descriptive Qualitative Study, International Journal of Nursing Studies. (2025) 10.1016/j.ijnurstu.2025.105249. 41138663

[38] Liu P. , Zhang Y. , and Meng Q. , The Mediating Effect Model of Nurses’ Sense of Perceived Overqualification, Professional Identity, and Job Burnout: A Cross-Sectional Study, International Nursing Review. (2025) 72, no. 3, 10.1111/inr.70090. 40781816

[39] Zhai S. , Dai C. , Liu Q. , Lu Y. , and Chen C. , Clinical Nurses’ Job Embeddedness and Its Relationship to Professional Identity, Organizational Climate, and Compassion Fatigue: A Structural Equation Model, Journal of Nursing Management. (2025) 2025, no. 1, 10.1155/jonm/3700369. PMC12436005 40959603

[40] Karadas A. and Çevik C. , Psychometric Analysis of the Quiet Quitting and Quiet Firing Scale Among Turkish Healthcare Professionals, Journal of Evaluation in Clinical Practice. (2024) 31, no. 2, 10.1111/jep.14136. PMC11938806 39279539

[41] Ejebu O. , Philippou J. , Turnbull J. et al., Coming and Going: A Narrative Review Exploring the Push-Pull Factors During Nurses’ Careers, International Journal of Nursing Studies. (2024) 160, 10.1016/j.ijnurstu.2024.104908. 39305682

[42] Yuan L. , Li Y. , Yan H. et al., Effects of Work-Family Conflict and Anxiety in the Relationship Between Work-Related Stress and Job Burnout in Chinese Female Nurses: A Chained Mediation Modeling Analysis, Journal of Affective Disorders. (2023) 324, 309–316, 10.1016/j.jad.2022.12.112. 36586602

[43] Yang S. , Tian L. , Fan J. et al., A Qualitative Research Exploring Clinical Nurses’ Experience With Nurse Employee Assistance Program Promoted by Hospitals in China, BMC Nursing. (2025) 24, no. 1, 10.1186/s12912-025-03739-w. PMC12400728 40890710

[44] Jalali R. , Fatahi S. , and Kolehjoubi A. A. , Quiet Quitting in Nursing: A Concept Analysis, BMC Nursing. (2026) 25, no. 1, 10.1186/s12912-026-04335-2. PMC12918568 41593580

